# Potential pharmacological approaches for the treatment of HIV-1 associated neurocognitive disorders

**DOI:** 10.1186/s12987-020-00204-5

**Published:** 2020-07-10

**Authors:** Amila Omeragic, Olanre Kayode, Md Tozammel Hoque, Reina Bendayan

**Affiliations:** Department of Pharmaceutical Sciences, Leslie Dan Faculty of Pharmacy, University of Toronto, 144 College Street, Room 1001, Toronto, ON M5S 3M2 Canada

**Keywords:** HIV-1, Microglia, Astrocytes, Neurons, Inflammation, Blood–brain barrier, Cytokine

## Abstract

HIV associated neurocognitive disorders (HAND) are the spectrum of cognitive impairments present in patients infected with human immunodeficiency virus type 1 (HIV-1). The number of patients affected with HAND ranges from 30 to 50% of HIV infected individuals and although the development of combinational antiretroviral therapy (cART) has improved longevity, HAND continues to pose a significant clinical problem as the current standard of care does not alleviate or prevent HAND symptoms. At present, the pathological mechanisms contributing to HAND remain unclear, but evidence suggests that it stems from neuronal injury due to chronic release of neurotoxins, chemokines, viral proteins, and proinflammatory cytokines secreted by HIV-1 activated microglia, macrophages and astrocytes in the central nervous system (CNS). Furthermore, the blood–brain barrier (BBB) not only serves as a route for HIV-1 entry into the brain but also prevents cART therapy from reaching HIV-1 brain reservoirs, and therefore could play an important role in HAND. The goal of this review is to discuss the current data on the epidemiology, pathology and research models of HAND as well as address the potential pharmacological treatment approaches that are being investigated.

## Epidemiology of HAND

### Prevalence of HAND in the era of cART

Human immunodeficiency virus type 1 (HIV-1) is the enveloped retrovirus responsible for the development of Acquired immunodeficiency syndrome (AIDS) in patients. The Joint United Nations Program on HIV/AIDS (UNAIDS) estimated that about 36.9 million people worldwide were living with HIV as of 2017, with approximately 21.7 million of that population receiving combinational antiretroviral therapy (cART) [1]. In addition to the development of AIDS, patients with HIV have been documented to have neurological complications as early as the late 80’s [2]. In their 1986 paper, Navia and Price first described the observed motor and behavioural deficits associated with AIDS as the “AIDS dementia complex”(ADC) [3]. Since then, three types of disorders have been recognized to define the observed neurocognitive deficits. In order of increasing severity, the terms are Asymptomatic Neurocognitive Impairment (ANI), Minor Neurocognitive Disorder (MND), and HIV-associated Dementia (HAD) [4]. In 2007, HIV Associated Neurocognitive Disorders (HAND) was proposed by the US National Institute of Mental Health panel to be used to define the spectrum of the neurological disease associated with HIV infection [4].

Determining the prevalence of HAND continues to be challenging as reported numbers are variable within the literature. In a study using patients from the Multicentre AIDS Cohort Study (MACS), Sacktor et al. observed a frequency of HAND amongst 364 HIV+ gay/bisexual men within the 3 year period of 2007–2008 of 33% (14% classified as ANI, 14% classified as MND and 5% as HAD) [5]. Other groups have reported the prevalence of HAND to be between 15 and 55% of HIV+ individuals [6–10]. Since the development of cART over two decades ago, the prevalence of HAND has not changed significantly but the severity of neurocognitive impairment has noticeably dropped. In the pre-cART era, approximately 16% of HIV+ individuals were also suffering from HAD [11] but more recent data has shown that after the introduction of cART, prevalence rates of HAD are within the range of 2–8% [6]. Although cART appears to be beneficial for attenuating the presentation of HAD, it does not yield positive results in the prevention of milder forms of HAND, as ANI prevalence has dramatically increased since the dawn of cART, and it is now the most common form of HAND [6, 11, 12].

Despite, the asymptomatic nature of ANI, it remains a concern as mild cognitive deficiency can quickly progress to more severe forms of HAND. In the MACS study the research team enrolled a group of 197 HIV+ participants on cART and assessed them every 2 years over a 6 year time period and observed an increase in HAND prevalence from 25 to 31% [5]. Within the study, 15% of the subjects experienced further cognitive decline (ANI to MND or HAD) over the 6 years, while 14% saw improvements in their HAND stage [5]. In 2014, another study demonstrated that an ANI diagnosis was associated with a higher risk of developing MND or HAD when compared to HIV+ patients with no signs of diminished cognition [13].

### Clinical aspects of HAND

#### Diagnosis and biomarker identification

A clinical diagnosis of HAND is reached based on the patient’s results on time-consuming neuropsychological tests assessing their abilities in memory, information processing speed, verbal language, attention and working memory, sensory perception, motor skills and executive functioning, where scoring at least one standard deviation below the age-appropriate mean in two categories or more is indicative of impairment [4]. The severity of impairment via neuropsychological testing cannot solely be taken into account when diagnosing patients. Healthcare professionals must also consider the degree of impact that these deficits have on a patient’s ability to function and the absence of any confounding factors that can otherwise explain the observed clinical symptoms [4, 14]. The typical diagnosis of ANI requires a below average performance on diagnostic tests, no negative impact on daily living, cognitive impairment not meeting the criteria for delirium or dementia, and the absence of other conditions that may cause cognitive impairment [4]. The classification of MND is similar except that the impairment must mildly impact the patient’s daily functioning through either self reported or observed deficiencies in work, homemaking, social interaction and mental acuity and typically results in a score between 0.5 and 1 on the Memorial Sloan Kettering scale. HAD patients typically score 2 or greater on the Memorial Sloan Kettering scale and have their daily functioning significantly hindered by their cognitive insufficiency [4].

At present, there are no validated biomarkers to diagnose HAND. Several potential markers have been identified in HIV+ individuals with neurological impairment, however most of them are typically associated with HAD rather than ANI and MND, the more prevalent forms of HAND [6]. The biomarkers can be divided into four general categories: (1) structural changes observed with neuroimaging, (2) markers of cellular or metabolic stress, (3) humoral markers of immune activation and (4) markers of neuronal injury.

In regards to brain structural changes, a multitude of studies conducting brain volumetric analysis with the use of magnetic resonance imaging (MRI) in the context of HAND are summarized in a review by Masters and Ances [15]. One of the more recent publications relates to a 2013 study including patients from the ANRS CO3 Aquitaine Cohort and reports that MND and HAD patients had lower gray matter and white matter volumes when compared to patients diagnosed with ANI [16]. Additionally, in a separate study, HIV+ individuals with quantifiable peripheral viral load displayed decreased subcortical and cerebellar gray matter volumes when compared to HIV+ subjects with undetectable viral load in the periphery [17]. The same study reported that HIV+ participants had enlarged ventricles and reduced putamina, hippocampi, nucleus accumbens, caudate nuclei, brainstems, thalami, total cortical gray matter and cerebral white matter compared to the HIV- control group [17].

The second type of biomarkers are classified as markers of cellular or metabolic stress. Elevated levels of Krebs Cycle substrates (acetate and citrate) in the cerebrospinal fluid (CSF) were found to be linked with worsening cognitive status in HIV+ patients [18]. A separate study published results indicating that reduced CSF concentrations of esterified cholesterols and sphingolipids in HIV+ patients increased the risk of cognitive decline [19]. Additionally, proton magnetic resonance spectroscopy was used to detect elevated choline compounds in the white matter of those diagnosed with ADC when compared to neuro-asymptomatic HIV subjects [20]. Other markers of cellular or metabolic stress include CSF heme oxygenase-1, CSF protein carbonyls, CSF 3-nitrotyrosine and brain inducible nitric oxide synthase (iNOS) [6].

Markers of immune activation are part of another class of potential biomarkers that include molecules such as neopterin. Neopterin is a metabolite of the guanosine triphosphate pathway in monocytes and macrophages and high levels have been reported in the CSF of HIV+ individuals [21]. Increased CSF neopterin is indicative of immune activation during HIV infection and thus significantly elevated concentrations suggests infection of the immune cells within the central nervous system (CNS). Neopterin levels are also correlated with the release of reactive oxygen species (ROS) by macrophages which is hypothesised to be a mechanism of neuronal cell damage leading to the symptoms of HAND in patients [21]. Another example of a marker of immune activation in the context of HAND is elevated C-C chemokine receptor type 2 (CCR2) on CD14+ and CD16+ monocytes [22]. CD14+CD16+ monocytes have a high vulnerability to HIV [23] and are considered to act as peripheral reservoirs for the virus. A 2014 study demonstrated that CCR2 was markedly increased in those suffering from HAND when compared to HIV+ patients with normal cognition and found that it did not change with viral load, CD4+ cell counts or with the use of cART [22]. CSF fractalkine, plasma sCD14 and sCD163, CSF osteopontin, CSF C-C motif chemokine ligand 2 (CCL2), brain interleukin-1β (IL-1β), and brain interleukin-10 (IL-10) are just a few of the numerous other potential immunological biomarkers for HAND under investigation [6, 24].

Finally, the last category of biomarkers are markers of neuronal damage. Neurofilament light chain (NFL) is well documented to be positively correlated with HAND [25, 26]. NFL is a surrogate marker for neuronal damage and elevated levels have been detected in the CSF of those with neurodegenerative disorders like subcortical vascular dementia and Alzheimer’s disease [27]. A recent study measured CSF NFL concentrations in 48 untreated HIV+ subjects not on ART and reported that NFL was significantly higher in patients with HAD compared to subjects with mild to no cognitive impairments [28]. It was also discovered that CSF NFL was positively correlated with plasma HIV-1 viral load and negatively correlated with peripheral CD4+ T cell count [28]. A separate study by Nitkiewicz et al. investigated the increased expression of complement proteins, such as complement 3 (C3), in human fetal astrocytes after exposure to HIV-1 [29]. The complement cascade is a critical factor in the pathogenesis of diseases in the CNS and C3 upregulation is indicative of neuronal injury and chronic neurodegenerative disorders. The upregulation of C3 is likely facilitated by the induction of interleukin 6 (IL-6) mediated by nuclear factor kappa-light-chain-enhancer of activated B cells NF-κB during HIV infection, as demonstrated in vitro [29]. Additional CSF markers for neuronal injury currently being investigated in the context of HAND are quinolinic acid, CSF total Tau concentrations, CSF soluble beta amyloid precursor protein and brain *N*-acetyl aspartate [6].

#### Risk factors

Risk factors for HAND have been well-documented and often overlap with observed comorbidities [6]. For example, one report documented that diabetes, high carotid intima media thickness, smoking, hypertension and dyslipidemia were highly prevalent in asymptomatic HIV+ subjects and subsequently were associated with lower cognitive performance [30]. Old age is also positively correlated with an increased risk of HAND. HIV+ individuals over the age of 50 years were twice as likely to develop HAD when compared to younger seropositive comparators, according to a study involving the Hawaiian Aging Cohort [31]. A more recent Japanese study also found that increased age was associated with a higher chance of developing MND and HAD [32]. Other risk factors reported in the literature include, sleep disorders such as sleep apnea, and co-infection with the Hepatitis C virus [6]. Substance abuse is another common risk factor, with the abuse of opioids, cocaine, marijuana, alcohol and methamphetamine being correlated with poor cognitive performance in HIV+ patients [33, 34]. Mental illnesses such as depression, schizophrenia and bipolar disorder are often observed in HIV+ patients and are strongly associated with medical nonadherence [35]. As a result, HAND patients may be at high risk of improperly using their antiretroviral (ARV) medications which could potentially lead to the exacerbation of their condition.

## Neuropathogenesis

### HIV infection and the CNS: cellular targets

The principal peripheral targets of the HIV-1 virion are circulating CD4+ T-lymphocytes and macrophages [7, 36]. The virus gains entry through interactions with the host’s CD4 surface protein and C-C chemokine receptor type 5 (CCR5) via its envelope glycoprotein [37]. Certain strains of HIV-1 can also infect cells using the C-X-C motif chemokine receptor 4 (CXCR4) as a coreceptor [38]. Due to the variable entry mechanisms used by the virus, it is suitable to distinguish HIV-1 into three groups. R5-tropic variants which make up the majority of HIV-1 and uses CCR5 as a co-receptor; X4-tropic variants which instead use CXCR4 to gain access to cells [39]; and finally dual-tropic HIV-1 strains which have the capability to use both co-receptors, yet the exact mechanisms on how it switches from one molecule to the other is not fully understood [40].

Within the first few weeks of infection, HIV-1 can enter the CNS [36]; viral RNA has been measured in the CSF of patients as early as 8 days after initial infection [41]. It is proposed that HIV-1 enters the CNS by crossing the blood–brain barrier (BBB) through the penetration of HIV-infected monocytes across the brain vascular endothelial cells or as cell free virions [42–44]. Approximately 5-10% of circulating monocytes are CD14 and CD16+, but that proportion increases during HIV infection [45, 46]. These monocytes have also been shown to be preferentially vulnerable to HIV-1 and thus are important to its associated neuropathogenesis [23]. Once infected, CD14+CD16+ cells present upregulated surface expression of the junctional proteins ALCAM and JAM-A and the receptor CCR2, which are required for CNS entry [43, 44]. Although baseline transport of normal human CD14+CD16+ cells was revealed to not be statistically different from infected cells, HIV-containing monocytes were shown to have a significantly higher transmigration across an in vitro human BBB model when compared to uninfected cells through a C-C chemokine ligand 2 (CCL2) mediated mechanism involving JAM-A and ALCAM surface proteins. Migration increased in a dose-dependent manner in the presence of CCL2, and antibody specific blocking of JAM-A and ALCAM fully inhibited BBB transport [44]. Blocking JAM-A, ALCAM or CCR2 may prove to be an effective prophylactic measure in the prevention of HAND in HIV+ individuals [43, 47].

In contrast, two studies have proposed that macrophages are not directly targeted by HIV-1 [48, 49] and that they do not contribute to virus production in vivo [48]. Instead, it was proposed that macrophages target HIV-infected CD4+ lymphocytes for phagocytosis, explaining the reason for viral DNA and proteins being detected in macrophages. This controversy led to a 2016 study by Honeycutt et al. which aimed to further identify the mechanism behind HIV-1 infection of the monocyte lineage. The study found no evidence of viral DNA in monocytes from HIV+ individuals [50]. The group then used a myeloid-only mouse model and observed that HIV-1 infection was observed to be sustained in macrophages in the brain and de novo HIV-1 synthesis in the absence of T-cells in vivo was demonstrated [50]. These results support the idea that HIV-1 spreading in the CNS can be facilitated by mature macrophages but suggests that initial CNS penetration by HIV-1 infected monocytes is not an entry mechanism of the virus, directly arguing against the predominant school of thought. This knowledge discrepancy highlights our need for further understanding of HAND pathogenesis in order to identify effective treatment approaches.

Cells of the monocyte–macrophage lineage are considered as a potential HIV-1 reservoir and play a role in virus dissemination. HIV-1 infected monocytes can circulate in the blood up to 3 days before migrating to various tissues such as the brain, where they differentiate into macrophages [51–53], and may infect microglial cells. Alternatively, but still not well understood, the cell-to-cell transfer of the virus from infected migrating CD4+ T cells into brain macrophages and microglia, and virus spreading between infected macrophages has been suggested [54, 55]. It has been shown that infected T-lymphocytes come in contact with macrophages leading to cell fusion and transfer of viruses to macrophages. The lymphocyte-macrophage cells further fuse with noninfected macrophages and produce highly virus-productive multinucleated giant cells, observed in lymphoid organs and CNS of HIV-1- infected individuals and SIV-infected macaques [54, 56–62]. Multinucleated giant cells, along with myelin pallor, activated microglia, reactive astrocytosis (proliferation and activation of astrocytes), and presence of microglial nodules are a hallmark of chronic HIV-1 infection [11, 63, 64].

**Fig. 1:**
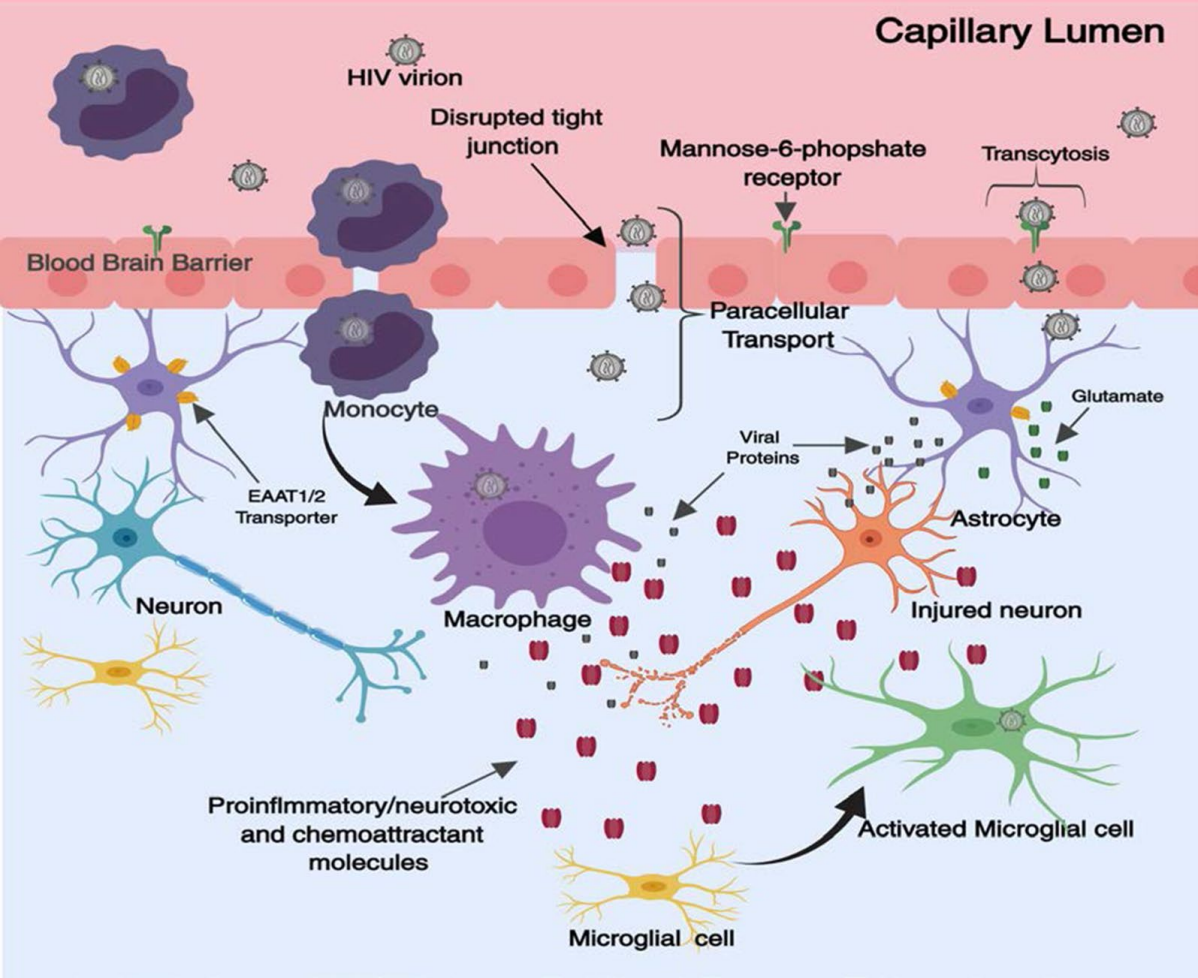
Neuropathogenesis of HAND. HIV-1 can enter the brain as a cell free virion or encased within infected monocytes or macrophages. Once in the CNS HIV-1 targets microglia, and to a lesser extent, astrocytes. Upon activation, these cells release numerous inflammatory markers (IL-1β, TNFα, CCL2 etc.) and can shed HIV-1 viral proteins (e.g. gp120, Tat). Chronic secretion of such factors which can exacerbate viral replication and pathogenic immune signalling ultimately leading to neuronal injury. HIV-1 infection in the brain may cause disruption of glutamate homeostasis leading to excitotoxicity (Figure adapted from Saylor et al. [6])

HIV-1 is also thought to be able to cross the BBB and enter the CNS as cell free virions [65]. Firstly, it may cross paracellularly into the CNS through a “leaky” BBB whose tight junctions have been compromised due to HIV-1 exposure [66–71]. Alternatively, a series of experiments involving in vivo and in vitro mouse models identified that the mannose-6-phosphate receptor expressed on brain microvessel endothelial cells can also mediate the transport of HIV-1 [65]. Ultrastructural studies revealed HIV-1 transport by the mannose-6-phosphate receptor in a transcytotic manner and transport was inhibited in the presence of mannose-6-phosphate, mannan (a plant polysaccharide), and mannose in the in vitro models. Transport was further reduced after exposure with endoglycosidase, an enzyme that cleaves high mannose oligosaccharide residues [65] (Fig. 1).

It should be noted that in addition to the BBB, the choroid plexus and meninges are also investigated as possible sites for virus dissemination to the CNS [72–74] as well as critical players in neuroinflammation [75, 76]. The CSF flow dynamics have also been recognized as a possible contribution of the CSF to disease pathogenesis. The potential for immune regulation in the CNS by the glymphatic system, an effector of neuroinflammation has also been suggested [77]. However, to keep the length of the manuscript concise, we primarily focused on the role of the BBB in HAND.

Following CNS infiltration, HIV-1 can infect the non-neuronal cells of the nervous system, namely microglia [78–80] and to lesser extent astrocytes [81–83]. Microglial cells initiate the brain’s innate immune response through their ability to congregate onto pathogens and destroy them. They express recognition receptors to detect pathogen-associated molecular signals and are capable of antigen presentation as well secreting cytokines and chemokines during an inflammatory response [79]. As microglia express both CD4 and CCR5 [78, 80], the mechanism by which they become infected with HIV is similar to that of leukocytes [80]. In the case of astrocytes however, this mechanism of HIV cellular entry cannot be applied. Astrocytes lack expression of the critical CD4 molecule that facilitates the infection of lymphocytes, microglia and macrophages but yet they have been shown to be susceptible to HIV and viral DNA and viral protein has been detected in post-mortem brain tissue from HIV+ patients [84–87]. Uptake of HIV-1 by astrocytes has been demonstrated to be facilitated by the human mannose receptor in a CD4-independent manner [88]. Expression of human mannose receptor rendered human astrocytes vulnerable to HIV-1 and siRNA silencing of the receptor blocked HIV-1 infection [88]. Despite the evidence in support of HIV-1 infection, controversial data exists that suggests that HIV-1 may not directly infect astrocytes after all. A 2001 in vitro study showed that there is a lack of intracellular mechanisms to restrict HIV-1 replication in primary human astrocytes [89], indicating that there was no evolutionary pressure for those cells in primates to defend themselves against HIV and related viruses and suggested that they are not main targets. Furthermore, Russel et al. suggested that the presence of HIV-1 DNA in cultured human astrocytes may be due to cell–cell interactions with infected macrophages [90]. Most recently, viral RNA and DNA were undetectable using single copy sensitive RNAscope and DNAscope on the astrocytes of aviremic HIV+ individuals on cART [91]. However, published data showed HIV-1 infection of astrocytes in HAND patients [92]. Together these conflicting reports highlight our limited knowledge in the understanding of HAND pathogenesis and present another barrier to effective treatment.

An additional group of cells susceptible to HIV-1 infection are the pericytes. Pericytes are known to wrap around the endothelial cells of the BBB with their cytoplasmic processes and facilitate interactions between endothelial cells. These cells are important for the formation, stabilization, and maintenance of the BBB [93]. Pericytes are known to express high levels of the CCR5 and CXCR4 receptors, as well as low-levels of the CD4 receptor [94], suggesting that pericytes can be infected by both X4 and R5 tropic HIV-1 strains. Recently, Bertrand et al. showed that pericytes can be infected with EcoHIV in vivo in mice, suggesting that pericytes could also be an HIV-1 target in the brain, however, the mechanism of HIV-1 entry into these cells remains unknown.

### HIV-1 toxicity

The direct neurotoxic effects of HIV-1 are demonstrated to be mediated solely by its viral proteins. There is little to no published research investigating if HIV-1 RNA or DNA is neurotoxic and directly contributes to neurodegeneration. At present, the evidence of HIV-1 induced neurotoxicity does not implicate direct contribution to toxicity by viral nucleic acids in the pathogenesis of HAND.

Available data generally proposes two main mechanisms of HIV-1-mediated CNS pathology leading to the development of HAND: (1) viral proteins from the HIV genome directly causing neurotoxicity, and (2) activation of microglia, brain macrophages and astrocytes in response to HIV-1 and secretion of a range of proinflammatory cytokines and neurotoxins [tumor necrosis factor-α (TNFα), interleukin-1β (IL-1β), interleukin-6 (IL-6), interleukin-8 (IL-8), arachidonic and quinolinic acids, platelet-activating factor, neurotoxic amines, ROS, nitric oxide (NO), glutamate, macrophage inflammatory protein 1α (MIP-1α), monocyte chemoattractant protein 1 (MCP-1) and growth-related oncogene α (GRO-α)], resulting in neuronal injury and death [95, 96].

#### HIV-1 tat

Trans-activator for transcription (Tat) is encoded by the *tat* gene. As one of HIV-1’s regulatory proteins, it is a key regulator for the transcription of proviral DNA to mRNA [97, 98]. Tat DNA sequences have been detected in the brain tissue of AIDS patients who suffered from dementia [99] and Tat mRNA along with protein were preferentially detected in brain tissues of patients suffering from HIV encephalitis (HIVE) [100]. The viral protein binds to the trans-activation response elements located at the 5′ ends of HIV-1 transcripts where it increases the activity of RNA polymerase II and thus greatly increases viral transcription [101]. In terms of its contribution to neurotoxicity during HIV-1 infection, Tat has been demonstrated to stimulate tumour necrosis factor alpha (TNFα) released by infected astrocytes resulting in neuronal death [102]. Other groups have also demonstrated Tat-mediated secretion of TNFα in glial cells [102–107] as well as increased release of IL-1β [103, 108, 109] and CCL2 [106, 110]. Studies have shown that Tat causes an upregulation of glial fibrillary acidic protein (GFAP) [111, 112] as well as decreased expression of two subtypes of excitatory amino acid transporters (EAAT1 and EAAT2) in mouse astrocytes [112]. EAAT1/2 are glutamate uptake transporters and their reduced expression may lead to an increase in glutamate concentrations in the CNS microenvironment, resulting in excitotoxicity [113]. Tat released by infected astrocytes has also been demonstrated to alter gap junction protein expression on the endothelial cells of the BBB leading to increased permeability [66] and Tat directly induces neuronal necrosis by disrupting mitochondrial function [104]. Recently, Tat was discovered to form a rigid multifibrillar structure by interacting with amyloid beta proteins in a mouse model that forms aggregates and mechanically disrupts the cell membranes of neurons and lead to the formation of pore [98].

#### Nef

Negative replication factor (Nef) is another regulatory protein of HIV-1 and its primary role is to decrease the transcription of various surface molecules and receptors in infected cells (such as MHC-I, MHC-II, CD3, CD4, CXCR4, CCR5, etc.) in order to avoid detection by the host’s immune system [114]. Nef has also been detected in astrocytes of patients with HIV encephalitis [85]. Data is limited in regards to its direct toxic effects, although Saribas et al. found that Nef-containing extracellular vesicles released by astrocytes induced oxidative stress in neurons and that Nef expression through adenoviral transduction in neurons led to the degeneration of axons [115]. It was also demonstrated that extracellular vesicles containing Nef were capable of suppressing action potentials in neurons, suggesting a role for Nef in HIV neurotoxicity. The mechanisms underlying these observations are still unclear.

Altered behaviour in rodents exposed to Nef has also been reported [116, 117]. For example, transgenic mice expressing Nef under the control of the *c*-*fms* promoter exhibited enhanced mania-like behaviour as demonstrated through enhanced locomotor activity, increased exploration times in an open field test, shorter periods of immobility in a forced swim test, and increased exploration in an elevated plus maze when compared to wild type mice [116]. These results support surrogate measures for manic behaviour and gives further insight into the role of Nef in behavioural deficits in those experiencing HAND. In parallel, these animals showed increased CCL2, decreased interferon alpha (IFNα) and disrupted dopamine levels in the striatum. A separate study investigating rats engrafted with Nef-expressing hippocampal astrocytes showed that these rodents had impairments in spatial and recognition memory as demonstrated by their failure in both novel location and novel object recognition tests [117].

#### Gp120

Glycoprotein 120 (gp120) is the HIV envelope protein that interacts with CD4 and CCR5/CXCR4 receptors to gain access into target cells [37]. Previous studies from our group have shown that gp120 stimulates the immune response causing the release of inflammatory, immune and oxidative stress markers both in vitro, in human and rodent astrocytes, in mixed glia, and in vivo [64, 118–120], in addition, it has also been demonstrated to be directly toxic to neurons of the CNS [121]. gp120 has been detected in macrophages and microglia of patients with HIV encephalitis [122, 123]. Studies examining the substantia nigra and caudate-putamina of rats showed that gp120 caused a loss in dopaminergic neurons [124]. The involvement of ROS in gp120-mediated neurotoxicity was further demonstrated in vivo and in vitro where gp120 preferentially induced apoptosis in dopaminergic neurons over non-dopaminergic neurons [125]. The gp120-induced apoptotic pathway is believed to use ROS intermediates as secondary messengers to increase intracellular Ca^2+^ [125], which may affect the Ca^2+^ balance in mitochondria, leading to programmed cell death in neurons through release of mitochondrial components such as cytochrome C and apoptotic protease activating factor 1 [126, 127]. Direct neurotoxicity by gp120 has also been reported by Chen et al. where rat cortical neurons were treated with gp120 and found that A-type transient outward K^+^ currents were enhanced in a dose-dependent manner [128]. A proposed mechanism of direct gp120 neurotoxicity includes mitochondrial dysfunction in exposed neurons [121].

Multiple groups have also shown that gp120 can reduce glutamate uptake in microglia and astrocytes by inhibiting glutamate transporter-1 (GLT-1) otherwise known as EAAT2, leading to excessive stimulation of neuronal N-methyl D Aspartate receptor (NMDAR) which results in an influx of Na^+^ and Ca^2+^ and subsequent cellular excitotoxicity [129, 130]. Gp120 has also been studied in the context of drug transporter and tight junction protein regulation at the level of the BBB [71, 131, 132] and in glial cells [119]. Data from these studies suggests that gp120 may have an impact on the integrity of the BBB and ultimately the disposition of certain pharmacological agents and endogenous molecules.

In parallel with gp120 neurotoxicity, behavioral dysfunction has also been reported. Morris Water Maze tests have shown that the V3 loop polypeptide of the gp120 protein can impair spatial memory in rats [133, 134] and electrophysiology tests revealed that exposure reduced long term potentiation in the CA1 region [133]. Deficits in locomotion have also been established in gp120 exposed rats, with exploration times observed to be significantly reduced when compared to controls [135, 136].

#### Vpr

Viral protein R (Vpr) is another accessory protein of HIV-1 and is important for the infection of cells of the monocytes-macrophages lineage and the nuclear localization of the pre-integration complex [137]. Using immunohistochemistry, Vpr has been detected in the basal ganglia and frontal cortices of patients with HIV encephalitis [138]. Vpr has also been reported to cause neuronal degeneration in vivo as well as to induce apoptosis in cultured human neuroblastoma cells [139]. Jones et al. demonstrated Vpr-induced neuronal loss which was mediated by p53 induction, cytochrome C release and activation of caspase 9 [139]. Microglial cells activated by Vpr were also found to release neurotoxins in a dose dependent pattern [139]. In a recent study, Vpr was shown to disrupt mitochondrial transport [140]. Vpr-treated neurons showed signs of accelerated aging through their increased expression of markers such as peroxisome proliferator-activated receptor-gamma coactivator 1 alpha (PGC-1α), X-gal, and p21^WAF−1^ [140]. HIV-1 infected microglia have also been known to express Vpr and release for subsequent uptake by proximal neurons [141]. A study by Rom et al. showed that human neuronal exposure to recombinant Vpr resulted in a sustained increase in intracellular Ca^2+^ which could impair glutamate signaling in neural cells as well as lead to the production of ROS [141]. Lastly, monocytes and macrophages infected with an HIV-1 variant that was incapable of producing Vpr released significantly less IL-1β, interleukin-8 (IL-8) and TNF-α when compared to HIV-1 wildtypes [142]. The observed reduction in apoptosis suggest a Vpr-dependent necrotic pathway mediated by proinflammatory molecules. An extensive 2016 review on the neuropathogenesis of Vpr conducted by James et al. further sheds light on the neurotoxicity of the viral protein [137].

### ARV treatment

HIV+ individuals have been treated with a combination of ARVs since their development in 1996 [143]. Patients on cART are required to take multiple therapeutic agents of different pharmacological classes designed to inhibit viral replication and entry. These classes include nucleoside reverse transcriptase inhibitors, non-nucleoside reverse transcriptase inhibitors, protease inhibitors, fusion inhibitors, entry inhibitors and integrase inhibitors [143]. Despite the complex pharmacotherapeutic intervention, HAND still prevails as a chronic condition that negatively impact patients’ quality of life, drug adherence and survival. cART is effective at decreasing viral load in the periphery of patients but the persistence of ANI and MND suggests that it could fall short on adequately controlling viral load in the CNS. A 2008 study followed 467 HIV+ individuals on cART (number of drugs included in their regimens ranged from 1 to 3) and assigned a CNS Penetration Effectiveness (CPE) score to each of the drugs within their regimen [144]. Scores from 0 (low CNS penetration) to 4 (high penetration) were given based on their chemical properties and observed concentrations in the CSF. The median CPE amongst the cohort was 1.5 and scores less than 2 were associated with an 88% increase in the odds of detectable CSF viral load [144]. Additionally, neurological impairment was negatively correlated to CPE scores in a cohort study involving 417 HIV+ participants on cART [145].

Recently, using a mouse model of HIV infection (EcoHIV) and the middle cerebral artery occlusion model, Bertrand et al. reported that HIV infection significantly increases the severity of ischemic stroke by affecting the BBB integrity and enhancing inflammatory response. Treatment of this mouse model with high CPE ART was more beneficial than low CPE ART in limiting tissue injury and accelerating post-stroke recovery, [146], thus establishing an additional beneficial effect of AVRs with a high CNS penetrance.

Improving CNS penetration of new ARV therapies may lead to a decrease in CNS viral load, but it poses an increased risk of neurotoxicity to patients [147]. The neurotoxicity of various ARVs was demonstrated in primary cultures of rat forebrain neurons where treatment with abacavir, efavirenz, etravirine, nevirapine, and atazanavir induced neuronal loss and damage. These compounds were highly toxic at levels above their current therapeutic concentrations [147], suggesting that increased CNS permeability of ARVs could be detrimental to patients. Clinical data on the use of efavirenz, for example, has shown that its use is associated with lower working memory, global functioning, processing speed, motor functioning and other signs of neurological decline [148, 149].

The lack of CNS penetration of ARVs may be due to the expression of membrane-associated drug efflux transporters at the BBB. Transporters such as P-gp are expressed on the luminal side of the vascular endothelial cells comprising the BBB and are responsible for pumping substances back into the blood to protect the brain from potentially harmful molecular entities [150]. Our group previously demonstrated that the ARVs abacavir, efavirenz, and nevirapine can activate the nuclear receptor Human Constitutive Androstane Receptor while the ARVs amprenavir, atazanavir, darunavir, efavirenz, ritonavir, and lopinavir can activate the Human Pregnane X Receptor in human brain microvessel endothelial cells [151]. These two nuclear receptors act as transcription factors and can regulate efflux transporters such as P-gp. For further details, please refer to our previous reviews [53, 152].

#### Approaches to eliminating the HIV-1 brain reservoir

Although antiretroviral therapy (ART) has significantly decreased the HIV-1 associated mortality and morbidity, the prevalence of HAND is continuing to increase, and conventional ARV regimens are insufficient to improve this condition. This is partly because many ARVs exhibit poor permeability across the BBB and blood-cerebrospinal fluid barrier (BCSFB), which results in low tissue bioavailability and subtherapeutic ARVs concentrations in the brain. The BBB and BCSFB are protected by brain microvessel endothelial cells and epithelial cells, respectively, and are known to physically and metabolically restrict ARV delivery into the brain [53, 153–155]. In addition to the presence of tight junctions, the permeability of ARVs into the CNS can also be highly regulated by the expression of drug efflux transporters such as P-gp, expressed at the BBB and BCSFB [53, 64]. As a result, the brain constitutes a reservoir for HIV-1 and presents a significant challenge to treating HAND. To bypass the effect of efflux transporters expressed at the BBB and BCSFB, and to increase the delivery of ARVs into the brain, multiple alternative approaches have been recently tested.

In particular, nanoparticle-based delivery of ARV drugs has recently been investigated. This system is known to protect ARVs from the effect of efflux transporters as well as from enzymatic and hydrolytic degradation, and can be used for a sustained release of therapeutics, in particular the novel integrase inhibitor, elvitegravir [156]. Although the data demonstrated that the encapsulated elvitegravir nanoparticle formulation improved the ability of the antiviral drug to cross the BBB model in vitro, in vivo data are needed in order to conclude that this strategy has a potential for therapeutic interventions in reducing HAND [156]. Agrawal et al. investigated the feasibility of developing a trojan horse prodrug that could simultaneously inhibit P-gp and have anti-HIV properties [157]. This could be a very promising approach which will need further investigation. In addition, Kaushik et al. developed a magnetic nanoformulation consisting of genome editing Cas9/gRNA bound with magneto-electric nanoparticles with the aim of targeting HIV-1 long terminal repeat, thereby stopping viral transcription and eradicating latent HIV infection. This is a very innovative approach deserving further investigation and that could potentially have clinical utility in the management of HIV infection of the brain [158].

## Animal models of HAND

Since the discovery of HIV-1, laboratory and animal models were rapidly implemented and developed to recapitulate the human disease. Animal models have helped to facilitate an in-depth understanding of HIV-1 while avoiding the use of human brain tissue which is challenging. Various animal models for investigating HIV-1 neuropathogenesis have been developed in order to investigate how systemic infection, immune activation and nervous system infection drive neuronal cell damage and death. A summary of various implemented models are outlined (Table 1). Several factors should be taken into account when aiming to reproduce HAND in animals. For example, the model should contain virus susceptible target cells, including CD4+ T lymphocytes, dendritic cells, monocytes and macrophages that display receptors and co-receptors for viral infection and possess the host cell machinery to complete the viral life cycle [37, 159]. Ideally animals should be infected through recognized sites of viral entry (e.g. blood, mucosal layers). Additionally, infection occurring for prolonged periods of time should be achieved in order to reflect the chronic nature of the disease [160]. Finally, viral infection should result in BBB impairment so that leukocyte transmigration can occur [161].

**Table 1:** Animal models of HAND

Name	Neuropathology	Neurological and behaviour deficits
Transgenic rodent models		
gp120 Tg mice	Astrogliosis, neuronal premature death, decreased dendritic arborization [166]	Age-specific memory deficits [167, 168]
GFAP-Tat Tg mice	Astrogliosis, neuronal premature death, increased monocyte and T-cell infiltration [169]	Tremor, ataxia, slowed cognitive and motor movements, seizures and hunched gestures [169]
Vpr Tg mice	Neurodegeneration [139, 170]	Hyper excitability, aberrant motor activity [139, 170]
*gag*-*pol* depleted *HIV*-*1* Tg mice	Reactive gliosis, vascular endothelial apoptosis [172]	Circling behaviour, hind limb paralysis [172]
Human reconstitution models		
HIVE mice	Neuronal cell death, astrogliosis, microglial activation [184–187]	Impaired working and spatial memory [184–187]
huPBL-HIVE mice	Astrogliosis, increased microglia activation, increased expression of IL-6, iNOS and IL-1β [188]	Not evaluated to date
hCD34+ cells and mouse lymphoid tissue repopulation	Reduction of neuronal soma, meningitis, astrogliosis, encephalitis [190, 191, 194, 195]	Not evaluated to date
BLT mice	Detectable viral load in the brain [196–199]	Not evaluated to date
Chimeric viruses		
EcoHIV	Detectable viral load in the brain, neuroinflammation, loss of MAP-2 and synapsin II staining [179, 337]	Impaired working and spatial memory [179, 181, 182]
Non-rodent animal models		
SIV infected macaques	Depletion of CD4+ cells, detectable viral load in the brain, neuroinflammation, neuronal loss [162]	Impaired performance in tasks assessing memory, fine/general motor skills, motivation, reaction time, spatial working memory [338]
FIV infected cats	Encephalopathy, reduced peripherical and motor neuron conductance [206, 207]	Aggression, loss of socialization, gait changes [206, 207]

Rodent models have been an instrumental tool used to study neuropathogenesis of HIV-1, as they offer several advantages such as: convenient handling, housing, and well characterized methods for manipulating their genome and affordability. Although HIV-1 does not naturally infect rodent cells, many approaches have been developed to circumvent this problem. For a more thorough review on animal models of HIV-1 please refer to the following publications [161, 162].

### Transgenic animals

One of the earliest approaches for modeling CNS infection was the generation of transgenic rodent models expressing human proteins necessary for HIV-1 replication. Initial attempts included transgenic rodents expressing human viral receptors CD4, CCR5 and CXCR4, however, all these were unsuccessful and with limited use [163–165]. The next approach was the implementation of transgenic animals expressing HIV-1 viral proteins in the brain. As mentioned earlier, several reports have demonstrated neurotoxicity associated with HIV-1 viral proteins. One of the original models investigating the role of viral proteins in the brain was the gp120 transgenic mouse model, where CXCR4 tropic gp120 was primarily expressed in astrocytes [166]. These mice developed age-specific memory deficits and the model helped to delineate cellular pathways involved in gp120 mediated neurotoxicity [167, 168]. Similarly, a Tat transgenic mouse model was also developed where Tat is expressed under the control of a doxycycline-dependent GFAP promoter, allowing for these mice to develop Tat-dependent brain pathologies such as astrogliosis, infiltration of monocytes/T-cells and premature death [169]. One of the more recent transgenic models is the Vpr transgenic mouse model, where Vpr is specifically expressed in myeloid cells in both the central and peripheral nervous systems [139, 170]. These mice acquire CNS abnormalities, and signs of peripheral neuropathy that are linked to mitochondrial dysfunction [171]. Other groups, including our own, have also demonstrated that a faster and more acute approach for investigating the role of these viral proteins in neuropathogenesis is direct injection of recombinant proteins into the brain [103, 118]. An alternative approach for viral protein expression in rodents through transgenic technology was the development of the transgenic rat model where *gag*-*pol* are deleted from the HIV-1 genome to render the virus non-infectious [172]. These rats develop behavioral and motor deficits [173].

### Chimeric viruses

Another innovative strategy for generating a small animal model of HIV-1 infection was demonstrated by Potash and colleagues who reengineered the virus in order to circumvent the obstacle of viral entry into murine cells [174]. This chimeric virus replaces gp120 with the murine leukemia virus gp80, facilitating entry into mouse cells [174]. Several studies have shown that mice infected with this chimeric strain display stable pro-virus in T-cells and macrophages, mucosal transmission of virus, normal CD4:CD8 ratios, a partially functional immune system and neurocognitive impairment [175, 176]. Our group along with others has demonstrated that intracranial (IC) administration of EcoHIV at a dose of 1x10^6^ pg p24 directly into the caudate putamen results in increased levels of several inflammatory genes [177–179]. Moreover, this dose leads to hippocampal dysfunction shown by defective long-term potentiation in hippocampal slices ex vivo and significant reduction in MAP-2 and synapsin II staining. Recently, several groups have investigated behavioural deficits and potential adjuvant therapies in this mouse model [179–182]. In one report, Kelschenbach and colleagues administered EcoHIV at a dose of 1 × 10^6^ pg p24 through intracranial (IC) injection directly into the caudate putamen of adult mice. The infected mice were subjected to radial arm water maze and cued-fear conditioning tests; deficits in both behavioural tests were observed starting at 19 days post infection [179]. Others have shown that inoculation with EcoHIV at a dose of 1 × 10^6^ pg p24 through intraperitoneal (IP) injection leads to the same behavioural impairments as early as 1 month post infection [180–182].

### Humanized mouse models

Humanized mouse models are becoming increasing popular for investigating interactions between the virus and host. Several variations of humanized mice have been developed through the use of severe combined immunodeficiency (SCID) genetic backgrounds. These mice have a mutant DNA-dependent protein kinase catalytic subunit, which causes the mice to lack functional B cells and T cells [183].

#### HIVE model

One of the earliest humanized models used for investigating neuroAIDS was the HIVE mouse model, where HIV-1 infected human monocyte-derived macrophages (MDMs) were injected directly into the basal ganglia of immunodeficient mice. Histopathological changes observed in these mouse brains included: formation of multinucleated giant cells, astrogliosis, microglial activation, and neuronal cell death. These mice also exhibited behavioural deficits [184–187].

#### huPBL-HIVE model

The next development of humanized mouse models aimed to examine the peripheral immunity in HIVE mice in order to get an in depth understanding of the adaptive immune system during HIV neuropathogenesis. Non-obese diabetic (NOD) mice were crossed with SCID background in order to enhance reconstitution with human peripheral lymphocytes (huPBLs). huPBL mice were synergistically injected in the brain with HIV-1-infected human macrophages [188, 189]. This model demonstrated the transmission of HIV-1 infected cells in the brain to human lymphocytes, the dissemination of virus throughout the blood, and the detection of HIV-1-specific cellular immune responses in the periphery [188]. A major limitation of this model is that these animals die within 4–5 weeks of engraftments due to human PBLs inducing graft-versus-host disease, where the human immune cells recognize the host mouse cells as foreign and attack them [188].

#### Human CD34+ cells and mouse lymphoid tissue re-population

The following series of models developed used NOD SCID mice crossed with interleukin-2 receptor g-chain (IL2Rg−/−) mice. The two versions of these mice were either engineered with partial deletion of IL2Rg−/− (NOG) [190] or complete deletion of IL2Rg−/− (NSG) [191]. IL2R is an essential protein for immune cell growth and maturation, and this mutation prevents the development of lymphomas. Therefore, the life spans of these mice are longer when compared to the standard NOD SCID mice. This model offered several other advantages compared to the HIVE model, for example, the ability to investigate peripheral and brain infection. Engraftment of NOD or NSG mice is performed by injecting human hematopoietic CD34+ stem cells derived from either cord blood, fetal liver or adult blood [191–193]. Human immune cells are present in numerous sites including the peripheral blood, liver, lung, vagina and rectum of these mice. NSG mice have been used as a useful model for investigating HIV-1-induced neuropathogenesis [194, 195].

#### BLT mice

The most recent humanized mouse model available is the bone liver thymus (BLT) mouse model. The approach for generating this model involves transplanting NOD SCID or NSG mice with human fetal thymus and liver cells following irradiation with human CD34+ cells from the same donor [196, 197]. The major advantage of these mice is that human T-cells develop within a human thymus, reflecting the clinical situation. Additionally, transmission of the virus can occur through the mucosal route. Studies have shown that BLT mice inoculated with HIV-1 have detectable levels of viral RNA and DNA in the brain [198, 199] and therefore, this could constitute another tool for investigating mechanisms and treatments for HAND.

It is important to note some of the limitations that exist in using humanized murine systems. For example, inconsistency in the amounts of grafted human cells, different mouse-to-human and human-to-mouse receptor-ligand interactions, varied populations of human and mouse macrophages and altered levels of infection dependent on human cell reconstitution and lack of significant mouse microglia. For more detail on brain pathologies of humanized mice please refer to [200].

### Non-rodent animal models

#### SIV

Simian immunodeficiency virus (SIV) infection is another closely related lentivirus which can be used as a tool to study HIV-1 pathogenesis. Over 40 strains of SIV have been discovered that naturally infect African non-human primate species [201]. The natural SIV infection of hosts does not typically lead to disease due to thousands of years of virus-to-host co-evolution, therefore these infected primates are not useful pathogenic models [162]. On the other hand, infection of Asian macaques with specific strains of SIV recapitulates many aspects of the disease in humans therefore, these have become the most widely accepted models for HIV/AIDS research. The macaque models have allowed for several advances in our knowledge of viral transmission, pathogenesis and latency, as explained in a review by Clements and colleagues [202]. The search for an effective vaccine and microbicides for prevention of HIV-1 has been extensively studied in this model. The most commonly used macaque species for AIDS are the rhesus macaque, the pig-tailed macaque and the cynomolgus macaque [162]. The use of SIV infected Asian macaques has undoubtedly provided tremendous insight into the pathogenesis of HIV/AIDS, however, it is important to note that there are fundamental differences between SIV and HIV-1 that limit the use of SIV-macaque models for the investigation of specific research questions. SIV is non-responsive to several drugs targeting HIV-1 protease, reverse transcriptase and integrase enzymes. Viral entry between the two strains can also differ as HIV-1 can in some cases utilize CXCR4, whereas SIV rarely uses this co-receptor but is capable of binding to other co-receptors that are not used by HIV-1 [203]. In order to circumvent this limitation, efforts have geared towards the developments of SHIVs, chimeric viruses which contain recombinants generated by replacing SIV viral genes such as rev, tat and env with corresponding HIV-1 genes.

Macaques have several advantages over small-animal models. SIV or SHIV infection of macaques reflects human infection in regard to cell tropism of viral infection, progressive depletion of CD4+ T cells and development of opportunistic infections typical of AIDS. Additionally, macaques and humans have a close phylogenetic relationship where many of the human genes controlling immune responses to HIV are similar to those in macaques. However, the use of this model as a research tool for many laboratories is limited due to high maintenance costs and genetic variability which can complicate studies. Nonetheless, despite these limitations, the SIV/SHIV model remains a precious tool to drive the HIV research field forward and ultimately bring us closer to a vaccine or cure.

#### FIV

Feline immunodeficiency virus (FIV) infection is a model which offers a natural approach to studying lentiviral-associated neuropathology. Similar to HIV-1, FIV infection results in an acute phase with minor symptoms, an inconstant latent phase and detrimental CD4+ T-cell depletion [204, 205]. Specific strains of FIV can lead to infection in the CNS and subsequent neuropathological changes similar to those evident in HIV+ patients [206, 207]. This model has served as a great tool for the development of several ARVs [208, 209].

## Potential pharmacological therapies for HAND

As the implementation of cART appears to do little to alleviate HAND, recent research has focussed on identifying new therapeutics to prevent cognitive decline in HIV-1+ individuals. Below is a summary of recent investigations. A chart displaying the disease models on which these compounds were tested can also be found (Table 2).

**Table 2:** Pharmacological agents investigated for HAND treatment

Drug class	Compound	In vitro system	In vivo system/clinical trials
Natural products	Resveratrol	Rat hippocampal slices exposed to Tat [105]*	Not evaluated to date
	Curcumin	Mouse microglia cultures and rat neurons treated with gp120 V3 loop [215]	Not evaluated to date
		Rat hippocampal slices treated with gp120 V3 loop [216–218]*	
		Mouse microglia exposed to gp120 [219]	
Antidiabetics	Insulin	Primary human microglia cultures infected with HIV-1 [229]	FIV infected cats [229]
	Rosiglitazone	Primary cultures of mixed rat glial cells exposed to gp120 [118]	Mice injected with gp120 ICV [118]
		Human brain endothelial cells exposed to Tat [226, 228]	MMP-9 deficient mice injected with Tat [227]
	15d-PGJ_2_	Human brain endothelial cells exposed to Tat [226, 228]	Not evaluated to date
	Pioglitazone	Primary cultures of mixed rat glial cells exposed to gp120 [118]	Mice injected with gp120 ICV [118]
	Troglitazone	Human brain endothelial cells exposed to Tat [226]	Not evaluated to date
	Fenofibrate	Not evaluated to date	MMP-9 deficient mice injected with Tat [227]
Glutamate modulators	Memantine	Not evaluated to date	gp120 transgenic mice [132, 238]
			HIVE SCID mice [239]
			SIV infected macaques [240]
	NitroMemantine	Not evaluated to date	gp120 transgenic mice [243]
	6-Diazo-5-oxo-l-norleucine	HIV infected microglia and macrophages [180]	EcoHIV infected mice [180]
	PPARγ agonists (rosiglitazone; pioglitazone)	Primary cultures of rat astrocytes exposed to gp120 [118]	Not evaluated to date
Statins	Simvastatin	Not evaluated to date	Rats ICV injected with gp120 [64]
	Unspecified	Not evaluated to date	HIV+ human patients on statin therapy [19]
Antiretrovirals	Maraviroc	Not evaluated to date	SIV infected macaques [257]
			HIV patients on stable cART [258]
			HIV+ patients with HAND [259, 260]
IFN therapy	IFNβ	Primary human fetal microglia infected with HIV-1 [270]	Transgenic gp120 mice [271]
		Rat cerebrocortical cultures exposed to gp120 [271]	
	B18R	Not evaluated to date	HIVE SCID mice [187, 278, 279]
Fumaric acid derivatives	Monomethyl fumarate	Primary human astrocytes co-cultured with HIV-1 transduced monocytoid cells [339]	Not evaluated to date
	DMF	HIV-1 infected human monocytes [281]	Not evaluated to date
		Primary rat neurons exposed to HIV-1 infect human monocytes [281, 340]	
		Human neuronal cells exposed to HIV infected human macrophages and neuroblastoma cells [285]	
Antibiotics	Minocycline	Not evaluated to date	SIV infected macaques [293]
			Rats injected with gp120 ICV [64]
			HIV+ patients with HAND [294, 296]
			HIV+ patients [295]
NSAIDs	Meloxicam	Not evaluated to date	Transgenic HIV-1 rats [298]
Steroid alkaloids	dCA	Primary human CD4+ T-cells infected with HIV-1 [301]	Tat transgenic mice [302]
		Human astrocytic cell line transfected with T*at* [302]	
Beta galactoside binding proteins	Galectin-1	Primary human microglia transfected with *Tat* [312]	Not evaluated to date
Cannabinoids	ACEA	Human brain microvascular endothelial cells with human astrocytes exposed to gp120 [319]	Not evaluated to date
	CP55,940	Human brain microvascular endothelial cells with human astrocytes exposed to gp120 [319]	Not evaluated to date
	WIN55,212-2	Human neurons exposed to gp120 [320]	Not evaluated to date
		Mouse prefrontal cortices exposed to Tat [321]	
	Anandamide	Mouse prefrontal cortices exposed to Tat [321]	Not evaluated to date
	AM1241	Primary human and murine neural progenitor cells exposed to gp120 [322]	Transgenic gp120 mice [322]
	Gp1a	Not evaluated to date	HIVE mice [323]
Others	Fluconazole and Paroxetine combination	Not evaluated to date	SIV infected macaques [331]
			HIV+ patients with HAND [332]
	Fluconazole	Mixed rat hippocampal cultures exposed to gp120 and Tat [331]	HIV+ patients with HAND [332]
	Paroxetine	Mixed rat hippocampal cultures exposed to gp120 and Tat [331]	HIV+ patients with HAND [332]
	Chloroquine	Not evaluated to date	Rats ICV injected with HIV gp120 [64]
			HIV+ adults on ART [335]
			Asymptomatic HIV infected adult patients not on ART [336]

*ex vivo model used

### Natural compounds

Studies investigating natural compounds as potential anti-inflammatory agents for the treatment of HAND are limited. Curcumin is the most extensively studied anti-inflammatory natural product in the context of HAND to date but there are numerous other compounds that may be viable candidates. A review by Kurapati et al. summarizes a number of plant derived compounds showing antiviral activity against HIV, however, none of these compounds have shown to target inflammation [210]. Additionally, a review by Shal et al. discusses the neuroprotective potential of several natural products in the context of Alzheimer’s disease [211]. The compounds discussed in both reviews with exception of resveratrol and curcumin have not been examined as treatment options for HAND and may be considered as candidates in the future.

#### Resveratrol

Resveratrol is a stilbene derivative that is synthesized in grape skin and is found in wine, with a significantly higher concentration present in red wines [212]. To the best of our knowledge, there is only one report investigating the anti-inflammatory effects of this compound in the context of HAND [105]. This study used an ex vivo system of rat hippocampal slices exposed to HIV-1 Tat. Treatment with resveratrol reversed the Tat induced expression of CCL2 and TNFα pro-inflammatory molecules. Western blot analysis showed an elevation of phosphorylated ERK2 in Tat-exposed slices, which was also reversed with treatment of resveratrol, suggesting that resveratrol attenuates inflammation by inhibiting the ERK1/2 pathway [105]. It is difficult to fully evaluate resveratrol’s potential in HAND therapy based on a single ex vivo study. A prior study showed its capability to inhibit TNFα and iNOS production in lipopolysaccharide-activated rat microglia [213] however, extensive investigation is required before considering resveratrol as a candidate therapeutic agent for HAND.

#### Curcumin

Curcumin is a bright yellow organic compound derived from turmeric, a member of the ginger root family [214]. Outside of its uses as a herbal supplement, cosmetic ingredient, spice, and food coloring agent, curcumin has also been investigated as a potential treatment for HIV-associated neuropathologies. A 2013 in vitro study examined the effects of curcumin on gp120 V3 loop-treated mouse microglia cultures and primary cultures of rat neurons [215]. The results showed that curcumin treatment led to an inhibition of gp120 V3-loop induced ROS production as well as TNFα and CCL2 mRNA upregulation [215]. Furthermore, the authors found curcumin to be protective in rat neurons by reducing apoptosis in gp120 V3 loop exposed cells [215]. Additionally, this study also demonstrated that curcumin treatment in rat neurons attenuated the HIV-1 gp120 V3 loop-mediated increased K^+^ current, and this was likely a mechanism mediating curcumin‘s anti-apoptotic effects [215]. In a separate study, an ex vivo system of rat hippocampal slices exposed to gp120 V3-loop curcumin treatment improved synaptic plasticity, as demonstrated by a decrease in Ca^2+^ concentrations in the hippocampal synaptosomes [216]. These results are consistent with older studies which reported that treatment of curcumin to gp120 V3 loop exposed rat hippocampal neurons resulted in attenuated neuronal injury, decreased caspase-3 expression and improved mitochondrial function and synaptic growth [217, 218]. Recently, in murine microglial cells exposed to gp120 in vitro, curcumin treatment was able to inhibit autophagy and inflammatory responses (e.g. CCL2, IL-17) [219]. The authors demonstrated that the anti-inflammatory actions of curcumin were mediated through the PI3K/AKT/IKK/NF-κB autophagic pathway [219].

Together, the present studies on curcumin’s neuroprotective properties in the context of HAND appear promising, however in vivo studies investigating this potential therapy are scarce and further research is required in order to move forward with this natural product. In addition to its anti-inflammatory effects, studies have also demonstrated curcumin’s anti-viral properties. A review by Prasad and Tyagi summarizes curcumin’s inhibition of HIV-1 proteins and enzymes such as HIV protease, HIV integrase and Tat [220].

### Anti-diabetic agents

#### PPARγ agonists

Peroxisome proliferator-activated receptor-gamma (PPARγ) is a ligand activated transcription factor that belongs to the nuclear receptors for steroid, thyroid hormones and retinoids and plays a major role in lipid and glucose regulation [221]. PPARγ agonists rosiglitazone and pioglitazone have been clinically proven as effective treatments for type 2 diabetes [221]. There is ample evidence suggesting that targeting the PPAR family is neuroprotective in several animal models of neurological disorders [222–224]. In the context of HIV-1, ample data from pre-clinical studies also supports PPARγ as an effective anti-inflammatory target in the brain [118, 225–228]. Studies have also demonstrated protective effects of PPARγ activation in reducing HIV-1 or Tat induced dysfunction in brain microvessel endothelial cells [226, 228]. Overexpression of PPARγ in a human brain microvessel endothelial cell line (hCMEC/D3) inhibited HIV-1 or Tat-mediated increases in IL-1β, TNF-α, CCL2 and E-selectin, which was partly mediated through inhibition of NF-кB transcriptional activity [228]. In this study, activation of PPARγ by an exogenous agonists (i.e., rosiglitazone) in hCMEC/D3 also protected against these responses, whereas, the antagonist of PPARγ reversed the protective effects [228]. The same group followed up with subsequent studies where, overexpression of PPARγ protected against increased matrix metalloproteases and proteasome activity, downregulation of tight junction proteins and increased monocyte migration in a co-culture model of hCMEC/D3 and astrocytes exposed to HIV-1 infected monocytes [226]. These results were further corroborated in an in vivo mouse model of Tat exposure through injection into internal carotid artery. Treatment with PPARγ agonist, rosiglitazone reduced Tat-induced BBB impairments, astrogliosis, and neuronal loss [227]. Our group has also demonstrated that both PPARγ agonists, rosiglitazone and pioglitazone, are protective against gp120 mediated inflammatory responses (IL-1β, TNF-α) in primary cultures of rat mixed glial cell cultures as well as in vivo in a rat model of gp120 exposure through intracerebroventricular injection [118]. Although the precise mechanism of how PPARγ agonists activate an anti-inflammatory response to HIV-1 remains unclear, our group has recently shown evidence of PPARγ agonists inhibiting NF-κB [118].Furthermore, targeting PPARγ has also proven to be anti-viral. Potula et al. have demonstrated that PPARγ activation by rosiglitazone resulted in suppression of HIV-1 LTR promoter activity and HIV-1 replication in MDMs through transrepression of NF-кB [225]. These results were corroborated in the HIVE model which demonstrated rosiglitazone-mediated suppression of viral replication in macrophages in brain tissues and 50% reduction in viremia in vivo [225]. More recently, our group has showed that treatment with PPARγ agonists rosiglitazone and/or pioglitazone in an EcoHIV mouse model effectively reduced HIV viral p24 protein burden in mice brains [178].

#### Insulin

Insulin is a hormone produced by the beta cells of the pancreas. The primary role of insulin is to help regulate blood sugar. Insulin is administered to diabetic patients who are unable to produce sufficient levels of insulin. Recently, the use of insulin as a therapeutic target for HAND has been explored. Mamik et al. demonstrated the anti-inflammatory effect of insulin in vitro and in vivo. In primary cultures of HIV-1 infected human microglia, insulin treatment reduced inflammatory genes CXCL10 and IL-6 as well as HIV-1 p24 levels in the supernatant [229]. In primary cultures of human neurons, insulin exposure prevented Vpr-mediated cell death [229]. In vivo, intranasal insulin treatment in FIV infected cats reduced the same markers (CXCL10, IL-6) and FIV RNA in the brain. Immunohistochemical analysis revealed diminished levels of glial activation and protection of cortical neurons. The neuroprotective mechanism behind insulin is not yet understood, but the authors hypothesize that insulin treatment regulates PPARγ expression in microglia and astrocytes, which may explain the reported observations. The molecular results were accompanied by functional improvement of neurobehavioral performance, including both motor and memory [229]. Furthermore, in an EcoHIV mouse model intranasal insulin beginning 23 days or 3 months post infection reversed neurocognitive impairments in mice. Insulin treatment also reduced HIV DNA in the brain, however, this was only achieved when treatment was initiated at earlier time points post infection (e.g. 23 days post infection) [182]. At present, there are at least two clinical trials that have been initiated for intranasal insulin for HAND treatment (Clinical trial IDs: NCT03081117 and NCT03277222) [230].

The prevalence of comorbid metabolic disorders is increasing as the HIV/AIDS populations ages. The risk of type 2 diabetes, stroke, hypertension and hyperlipidemia can be further exacerbated by cART [231–233]. Type 2 diabetes has been reported to be an established risk factor for HAND and other neurological disorders such as Alzheimer’s. Therefore, these compounds seem to be the most promising HAND treatments, as they could be used to treat not only the neurocognitive aspect but also the metabolic comorbidities. Additionally, as they are already in clinical use this could allow for relatively quick translation for use as therapy for HAND.

### Glutamate modulators

Glutamate is an abundant excitatory neurotransmitter present in the brain which plays a critical role in synaptic plasticity. Under homeostatic conditions, glutamate is important for cognitive functions such as learning and memory. Glutamate is cleared from the extracellular space by specific uptake transporters expressed in neurons and glial cells. EAAT2 in humans or GLT-1 in rodents is mainly expressed in astrocytes and is the primary transporter responsible for glutamate clearance [234]. Once glutamate enters astrocytes it is converted into glutamine and released to the extracellular space where it is subsequently taken up by neurons which convert it back into glutamate which plays a role in neurotransmission. Impaired glutamate homeostasis can lead to glutamate excitotoxicity which is defined as excessive activation of receptors such as NMDA, leading to increased intracellular Ca2+ levels and subsequent activation of proteases and endonucleases that can damage cellular components. Excitotoxicity has been proposed to contribute to several neurological diseases including HAND [235]. Studies have shown that HIV-1 infected individuals have five-fold greater levels of glutamate in the CSF when compared to healthy controls [236]. Another report investigating patients receiving combinational ARV therapy, observed that those diagnosed with HAND had increases in CSF glutamate levels compared to the individuals without neurological impairments [237].

As ample evidence has demonstrated the importance of glutamate regulation in the context of neurological disorders including HAND, several groups have employed different strategies to modulate glutamate excitotoxicity. The use of memantine, an uncompetitive NMDAR antagonist which has been clinically validated as a treatment for moderate to severe Alzheimer’s Disease, is one such example. Numerous pre-clinical studies have demonstrated neuroprotective effects of memantine in various HAND models; gp120 mouse models [132, 238], HIVE SCID mice [239] and SIV-infected macaques [240]. Unfortunately, the benefits of memantine failed to translate in clinical trials in patients with HAND [241, 242]. More recently, Nakanishi and colleagues developed a series of improved derivatives of memantine, termed NitroMemantines, which allosterically inhibit NMDAR activity through an adducted nitro group that reacts with redox modulatory sites on the receptor. Treatment with NitroMemantine protected against gp120 induced neuronal damage and synaptic loss in the hippocampus of gp120 transgenic mice [243]. Although memantine was unsuccessful in improving neurocognitive function in HAND patients, it is possible that NitroMemantine could result in a better outcome for these patients, as it preferentially inhibits extra synaptic NMDARs, which are relevant to glutamate mediated excitotoxicity [244].

Other strategies that have been investigated for modulating glutamate homeostasis include regulation of enzymes that are responsible for generating glutamate [245] or regulation of the glutamate transporters themselves [118, 246]. Erdmann et al. showed that inhibition of glutaminase by glutaminase specific small molecule inhibitors or glutaminase specific siRNA were successful in preventing increased glutamate production in vitro by HIV-1 infected macrophages [245]. Unfortunately, there are no clinically available glutaminase inhibitors that penetrate the CNS. More recently, Nedelcovych et al. 2017 used glutamine antagonist 6-diazo-5-oxo-l-norleucine and successfully showed that this compound attenuates glutamate synthesis in HIV-infected microglia/macrophages and prevents spatial memory deficits in EcoHIV infected mice [180]. Although these results are exciting, this compound cannot be used clinically due to associated peripheral toxicities. The same group synthesized several prodrugs of 6-diazo-5-oxo-l-norleucine which aimed to enhance brain delivery while limiting peripheral exposure [180]. Given the efficacy of glutamine antagonists in neuroprotective effects, these novel compounds should be considered for clinical testing in patients with HAND. Another strategy that has been investigated is to specifically target the transporters for glutamate uptake, such as EAAT2/GLT-1. A series of reports have demonstrated that this transporter is dysregulated in the context of HIV-1 associated neurological complications, likely mediated by HIV-1 viral proteins such as gp120 [118, 129, 130]. Our group has shown that treatment with PPARγ agonists (rosiglitazone or pioglitazone) reverses the gp120 mediated downregulation of GLT-1 in primary cultures of rat astrocytes [118]. Previous bioinformatic analyses revealed that there are at least 6 putative consensus PPAR response element sites in the promoter region of the EAAT2 gene and in vitro treatment with rosiglitazone increased promoter activity, therefore PPARγ may be regulating GLT-1/EAAT2 at the transcriptional level [247].

The importance of glutamate involvement in HAND has been made abundantly clear, establishing it as an important component that warrants further study. Attempts to modulate glutamate response to alleviate HAND symptoms and pathogenesis have thus far been unsuccessful in the few existing clinical studies. However, with newly developed promising compounds, such as NitroMemantine or PPARγ agonists, research can now offer new candidates for human studies.

### Statins

Statins make up a class of drugs capable of reducing cholesterol production in the liver by inhibiting the enzyme HMG-CoA reductase [248]. They are primarily used for the management of hypercholesteremia and the reduction of cardiovascular risk in patients. Aside from their role in limiting blood cholesterol, statins have also been shown to exhibit anti-inflammatory properties although the investigation of their effects on HIV-1 associated brain inflammation is limited. In a study previously performed by our group examining the anti-inflammatory properties in a rat model of gp120 induced neuroinflammation, simvastatin reduced enhanced brain expression of iNOS and IL-1β [64]. Yadav and colleagues demonstrated the abilities of atorvastatin and simvastatin in modulating the function and phenotype of human peripheral blood mononuclear cells. Treatment with a combination of atorvastatin and simvastatin reduced the proportion of CD16+ monocytes in PBMCs and purified monocyte cultures [249]. This is relevant as CD16+ cells are highly susceptible to HIV-1 and their migration to the CNS is vital to HIV-1 neuropathogenesis [250]. Treatment with simvastatin alone was also observed to reduce monocyte chemotaxis through inhibition of CCL2 secretion [249].

Despite the encouraging data from in vitro studies, the efficacy of statins has not been as promising in clinical studies. Bandaru and colleagues found no association with worsening or improved cognitive status in 19 HIV+ subjects actively on statin therapy [19]. This aligns with a previous pilot study in HIV+, ARV naïve males where treatment with atorvastatin failed to reduce HIV-1 RNA levels in the CSF and there were no changes in white blood cell counts or neopterin CSF concentrations [251]. However, there is a need to properly evaluate their therapeutic potential in the context of HAND, as a comprehensive review by van der Most and colleagues described the neuroprotective mechanism of statins in Alzheimer’s, Parkinson’s, Multiple Sclerosis and strokes [252] which may translate into HAND treatment.

### ARVs

#### Maraviroc

Maraviroc (MVC) is an approved ARV that acts as a CCR5 receptor blocker to prevent the entry of HIV into target cells [253]. Its relatively high CNS penetration and low neurotoxicity [147, 254, 255] has made MVC an attractive compound for the treatment of HAND. Early in vitro studies have shown MVC to inhibit the migratory response of macrophages to CCL2 [256], suggesting anti-inflammatory actions of this CCR5 antagonist. SIV infected monkeys treated with MVC monotherapy for 5 months had decreased viral loads in the CNS as proven by an observed reduction in viral RNA and DNA in the basal ganglia [257]. Additionally, treated macaques had lower expression of TNFα, CCL2, and reduced macrophage activation in the brain [257]. Amyloid precursor protein levels were also reduced further supporting the concept that MVC is neuroprotective in an SIV macaque model [257].

The encouraging results from in vitro and in vivo studies combined with the fact that MVC is already approved for human use allowed for pilot clinical trials to be performed relatively quickly. A small, single arm, open label study intensified the cART regimen of 12 stable HIV+ patients with undetectable plasma viral RNA but detectable monocyte HIV DNA. These patients were subjected to MVC treatment for 24 weeks and their monocyte HIV DNA, circulating CD16+ monocytes levels and neuropsychological performance were monitored [258]. Flow cytometry and RT-PCR results demonstrated a reduction in both monocyte HIV DNA and plasma CD16+ monocytes content in study participants. MVC treatment improved neuropsychological test scores in half of the participants who had previously showed evidence of mild to moderate cognitive impairment [258]. Further pilot studies with MVC intensified cART therapy in patients with HAND show similar results. For example, in a separate single arm trial, HIV+ participants with associated cognitive deficits and plasma viral suppression were switched from their cART regimen comprised of tenofovir, emtricitabine, and efavirenz to one containing abacavir, lamivudine and MVC for 48 weeks. These patients underwent neuropsychological testing as well as had their blood and CSF analysed. There was no significant difference in improved global deficit scores or CSF inflammatory markers with the exception of reduced CSF TNFα [259]. More recently, a randomized controlled clinical study performed in HIV+ patients diagnosed with HAND compared the efficacy of MVC intensified cART on cognition to participants’ existing cART treatment for 12 months [260]. Results from this study showed an improved global neurocognitive performance in the MVC group over the control but the authors could not detect metabolic differences in the brains of subjects between the two groups nor could they detect significant treatment-related changes in neopterin or β2-microglobulin levels in the CSF, two molecules associated with neurocognitive impairment in HAND [21, 261].

The evidence in support of MVC’s beneficial effects on cognition in HAND patients is a positive first step into investigating its use as a therapeutic option, but the current published studies are plagued by poor sample sizes. Also, to the best of our knowledge, there is only one study conducted that was randomized and contained a control group, which is clearly insufficient to fully evaluate the potential of MVC. The studies also seem to be lacking in information on the effects of MVC on chemokine and cytokine levels in those with HAND in order to evaluate the efficacy of MVC in the context of HAND pathogenesis. Further clinical studies with larger sample sizes and more sensitive assays to detect changes in inflammatory markers must be implemented in order to make an informed evaluation of the therapeutic potential of MVC.

### Type I interferon modulation

Type I interferons (IFN) are protein cytokines released by host immune cells in the presence of pathogens such as bacteria, viruses and tumor cells. Specifically, type I IFNs (IFNα and IFNβ) are secreted during viral infections where they bind to Interferon-alpha–beta receptors (INFARs) to upregulate antigen presentation and to express proteins to inhibit viral replication. Studies have shown that type I IFNs are implicated in both attenuating and exacerbating neuroinflammation triggered by HIV-1 infection. As a result, modulation of type I IFNs may be a key factor in treating HAND. A review on type I IFNs and their responses by Donlin and Ivashkiv provides great detail on this topic [262].

#### Interferonβ

Cocchi and colleagues showed that INFβ exposure in human microglia cell cultures induces the secretion of β-chemokines CCL3, CCL4 and CCL5 [263]. These endogenous CCR5 receptor ligands have been shown to inhibit HIV-1 infection and the progression to disease [264] and therefore make IFNβ a potential therapeutic option. IFNβ has already been shown to facilitate anti-inflammatory responses by down-regulating cytokine expression in CD4+ lymphocytes (IL-2, IFNγ, and IL-12), suppressing IFNγ-triggered iNOS expression by glial cells, and inhibiting the induction of MHC antigens [265–267]. Specifically, evidence supporting the neuroprotective roles of CCL4 and CCL5 against gp120-mediated toxicity in rat and murine neuronal cultures have been previously reported [268, 269]. Furthermore, INFβ inhibits HIV-1 infection in primary cultures of human fetal microglia [270]. Infection was enhanced when the cells were co-administered with anti-CCL4/5 antibodies, suggesting that INFβ indirectly supresses HIV-1 infection by upregulating β-chemokines [270].

Recently, the protective effects of IFNβ in HIV-1 related neuronal injury was investigated. In vitro and in vivo experiments with IFNβ were performed in rat cerebrocortical cultures containing astrocytes, neurons and microglia and in gp120-expressing transgenic mice [271]. In their in vitro experiments, recombinant murine IFNβ (mIFNβ) prevented gp120-mediated neuronal death in rat mixed cultures. Interferon-stimulated gene and protein expression (CCL5, CCL4, CCL3, CXCL10) were increased following IFNβ treatment. Neutralizing antibodies against each interferon-stimulated chemokine revealed that CCL4 is responsible for mediating the neuroprotective effects of IFNβ [271]. In vivo, gp120 transgenic mice treated with intranasal mIFNβ had increased CCL4 mRNA expression, higher levels of MAP-2 and synaptophysin and lower levels of Iba1 when compared to vehicle treated mice, demonstrating that IFNβ mediated neuroprotection [271]. This supports the results of an earlier in vivo study where type I IFN receptor knock out mice injected with EcoHIV experienced worse progression to disease when compared to wildtype, suggesting the involvement of type I IFN signalling in restricting HIV-1 infection and pathogenesis in the mice brains [177].

IFNβ is proposed to inhibit HIV-associated neuronal damage by binding to INFAR1/2 and increasing the expression of interferon-stimulated genes, particularly CCL4, to inhibit dendritic and synaptic injury. Current available studies with IFNβ in the context of HAND illustrate the signalling protein as a promising therapeutic option however there is a lack of behavioural studies. IFNβ is already FDA-approved for the management of multiple sclerosis [272] and therefore remains one of the most promising options for treating HAND.

#### B18r

Interferon alpha (IFNα) has been implicated in contributing to the neuropathology observed in HAND patients as high CSF concentrations of this cytokine have been reported to be positively correlated with cognitive impairment in HIV+ individuals [187, 273–275]. It is thought that IFNα leads to dendritic simplification through a mechanism mediated by INFAR and NMDA receptors, with the 2A subunit of the NMDA receptor playing a vital role in IFNα-mediated neurotoxicity [276]. In the endeavour to prevent IFNα related neurocognitive decline in HIV+ patients, research has shifted towards controlling IFNα with B18R, a recombinant protein originally expressed in vaccinia virus that is a type I IFN receptor capable of inhibiting type I IFNs in a wide variety of species [277]. In a HIVE/SCID mouse model of HAND, B18R was investigated for its BBB permeability, ability to neutralize IFNα and subsequently attenuate histopathological complications. HIVE/SCID mice administered IP with B18R three times daily for a total of 10 days [278]. Immunohistochemistry staining of brain tissue detected B18R, confirming its ability to cross the BBB. RT-PCR analysis also demonstrated the inhibitory effect of B18R on IFNα signalling by showing a downregulation of IFNα stimulated genes such as ISG15, IFNA4, and Ifrng15 [278]. More recently, Koneru et al., 2018 also investigated the neuroprotective effects of B18R in the HIVE/SCID mouse model. In this study, subcutaneous treatment twice daily with B18R in combination with a common cART regimen (atazanavir, tenofovir, and emtricitabine) prevented astrogliosis, the presence of mononuclear phagocytes and preserved staining of MAP2. Behavioural deficits in memory were investigated and results showed that treatment with B18R alone improved HIV-1 induced decrease in discrimination indexes [279]. Although the behavioural studies are limited, the results are promising and the authors plan to move forward with a Phase I clinical trial for B18R [279].

### Others

#### Dimethyl fumarate

Dimethyl fumarate (DMF) is a methyl ester of fumaric acid and is hydrolysed to its active form monomethyl fumarate. Currently, it is approved for the treatment of psoriasis and multiple sclerosis in adults [280]. Although its precise mechanism of action is unknown, DMF has shown to be immunomodulatory and anti-inflammatory, making it a potential agent for targeting the neuroinflammation observed in HAND. A 2011 study found that direct treatment with DMF was able to reduce HIV-1 reverse transcriptase activity in human monocytes infected with HIV-1 [281]. In addition, when primary cultures of rat neurons were exposed to HIV infected human macrophages, DMF treatment increased neuronal survival rates, likely due to their role in suppressing HIV replication and neurotoxin release. CCL2 release by infected monocytes was attenuated by DMF and its metabolite, and DMF was also shown to inhibit TNFα production and NF-κB signalling, suggesting that DMF’s effects may be due to their modulation of inflammatory pathways [281].

One of the most recent studies investigating DMF and its possible role in treating HAND looks at lysosomal dysfunction and the release of neurotoxic cathepsin B from infected macrophages. Increased cathepsin B release has been evidenced in HIV-infected macrophages and promotes neuronal apoptosis [282–284] and infected macrophages treated with DMF showed decreases in HIV-1 replication, cathepsin B secretion and ROS/RNS production [285]. DMF has consistently shown evidence of reducing HIV-1 replication in macrophages and inhibiting the release of neurotoxic compounds in vitro, but conflicting results on neuronal cell viability prevents a definitive conclusion of DMF’s role in HAND therapy. The discrepancy may be due to species differences between human and rats or to the differences in cell types used (primary neurons vs neuroblastoma cells). To the best of our knowledge, in vivo studies using fumaric acid derivatives for HAND treatment do not exist and further work is needed to confirm its therapeutic potential.

#### Minocycline

In the search for alternative treatments for the associated neuroinflammation in HAND, the potential efficacy of minocycline has been examined. As a second generation tetracycline antibiotic, minocycline has shown promise as an inhibitor of microglia activation; in vitro models of brain inflammation demonstrated its neuroprotective effects through reducing expression of pro-inflammatory cytokines [286, 287], inhibition of apoptotic cell death [288–290], nitric oxide synthesis [291]. This, coupled with its highly lipophilic profile that allows it to easily cross the BBB [292], renders minocycline as a promising candidate for HAND therapy. Early work investigating the neuroprotective impact of minocycline on HIV-related neuroinflammation used an SIV macaque model of neuroAIDS [293]. Daily oral administration of minocycline to SIV infected macaques prevented neuronal injury, decreased astrogliosis and microglial activation [293]. Additionally, further evidence to support the anti-inflammatory effects of minocycline in the context of HIV-1 was reported by our group, where intracerebroventricularly administered gp120 rats treated with minocycline had reduced protein and mRNA levels of inflammatory markers [64].

Results from the in vitro and in vivo studies encouraged the investigation of minocycline in clinical trials for HAND therapy. A randomized double blind, placebo-controlled study treated HIV+ participants with cognitive impairment with oral minocycline every 12 h for 24 weeks where its safety and efficacy was assessed through frequency of adverse events and changes in neuropsychological test composite z scores(NPZ-8) [294]. The authors found no change in NPZ-8 over 24 weeks between the treatment group and the control group. Similar results were observed in a later clinical trial in 2013 where HIV+ participants with low CD4+ cell counts were given oral minocycline every 12 h and had their cognitive function measured using the Uganda Neuropsychological Test Battery Summary Measures and the MSK scale [295]. Minocycline was shown not to be effective for improving cognitive function. A year later, minocycline’s effects on CSF markers of neuronal injury, inflammation, neurotransmitter levels and oxidative stress were tested using the same participants from the previous 2013 clinical study. CSF concentrations of numerous markers such as TNFα, IL-6, ceramides, quinolinic acid and glutamate was measured after 24 weeks while participants were receiving oral minocycline [296]. Minocycline was only found to significantly reduce CSF ceramide, a marker of oxidative stress. This study further demonstrated minocycline’s ineffectiveness as a therapy for HAND clinically, despite previous animal studies suggesting the opposite.

The discrepancy between the effects of minocycline in SIV encephalitis models and HIV+ patients has yet to be explained. A recent study reports that viral load and neuronal damage were reduced with minocycline in a SIV neuroAIDS model using macaque monkeys [297]. Perhaps the interspecies differences between humans and macaques are great enough to demonstrate different responses to minocycline, which may be the explanation for the contradictory data. Furthermore, the macaques studied in the SIV research models were studied from a maximum of 24 weeks after being inoculated with SIV, whereas participants in clinical trials may have been living with their infection for years prior to their involvement in studies. Based on the data from the clinical studies, minocycline does not appear as a therapeutic option for HAND.

#### Meloxicam

Meloxicam is a nonsteroidal anti-inflammatory drug, meloxicam reduces prostaglandin production, and thus inflammation, by selectively inhibiting COX-2 enzymes. Used in veterinary and human medicine, it has been FDA approved for the treatment of osteoarthritis. To the best of our knowledge, only one study investigated the anti-inflammatory effects of meloxicam in the context of HIV-1 associated brain pathology. Transgenic HIV-1 female rats were administered meloxicam daily and underwent a battery of behavioural tests. The purpose of the study was to verify if HIV-1 proteins can cause depressive-like behaviours in rats via alterations in neuroinflammation and cell proliferation, and if the treatment with meloxicam can reverse the observed behavioural patterns. The authors found that transgenic mice had increased CCL2 gene expression and displayed depressive like behaviours [298]. Treatment with meloxicam reduced CCL2 expression but was not able to attenuate depressive behaviours. From this study alone, meloxicam’s anti-inflammatory profile is somewhat promising but it is unclear if meloxicam will be a viable treatment option, continued research will provide further insight on the role of meloxicam in HAND therapy.

#### Didehydro-cortistatin A

Didehydro-cortistatin A (dCA) is a steroidal alkaloid analog isolated from *Corticium simplex*, a marine sponge [299]. Initially discovered to have anti-proliferative effects [300], dCA was also found to inhibit Tat-mediated HIV-1 replication in CD4+ T-cells from infected patients [301]. A study published in 2015 investigated the therapeutic potential of dCA in a *tat*-transfected human astrocytic cell line as well as its effect on cocaine reward in Tat transgenic mice [302]. In the Tat-expressing astrocytic cell line, qPCR analysis revealed an increase in TNFα and IL-1β gene expression and surprisingly, a decrease in CCL2 gene expression, which were all returned to baseline upon dCA treatment [302]. Additionally, extracellular uptake of Tat into human glial cell lines was reduced upon dCA treatment. In vivo, dCA was able to efficiently cross the BBB and its treatment reversed Tat mediated potentiation of cocaine reward in Tat transgenic mice [302]. This is thought to be of clinical importance as substance abusers make up a significant population amongst HIV+ individuals and previous studies have shown that Tat and cocaine can collectively augment neuronal damage [303–305].

Other studies examining dCA as an inhibitor of neuroinflammation in HAND are scarce. Investigations primarily look at dCA as a potential therapy to manage HIV-1 infection in patients due to the evidence of dCA being a potent inhibitor of Tat, and by extension, HIV-1 replication [306–308]. It is proposed that dCA inhibits Tat by binding to its basic domain and blocking its interaction with viral RNA [301]. With a lack of available studies on the topic, it is unclear how likely dCA will function as a therapeutic agent. Although it was shown to reduce Tat-mediated inflammation in vitro, there is no in vivo data to corroborate this. Additionally, there are several other HIV-1 proteins other than Tat that can initiate an immune response in the brain. With that being said, there is still much to learn before an informed assessment of dCA’s potential can be made. However, with its ability to cross the BBB, its lack of toxicity in astrocytic cell lines, and its modulation of inflammatory cytokines further testing of dCA is needed.

#### Galectin-1

Galectin-1 is part of a large family of β-galactoside-binding proteins. Evidence suggests that it has a role in modulating cell to cell interactions and its expression is correlated with neuro-regeneration [309, 310]. Galectin-1 is thought to be a key modulator of CNS homeostasis, with specific effects attenuating proinflammatory cytokines and oxidative stress markers while enhancing expression of anti-inflammatory cytokines such as TGF-β and IL-10 in microglia [311]. In the context of HIV-1 associated brain pathology, a recent study showed that galectin-1 treatment in HIV-1 Tat transfected primary human microglia exposed to TNFα was able to inhibit nitric oxide production and ROS/RNS activity, likely through limiting the availability of l-arginine in the iNOS-mediated nitric oxide production pathway, and by inhibiting the expression of iNOS [312]. Furthermore, galectin-1 promoted the protective M2 phenotype in the transfected microglia [312]. To the best of our knowledge, this is the only published study investigating the effect of galactin-1 treatment in the context of HIV-1. Further in vitro and in vivo studies are necessary for a more complete understanding of galectin-1’s potential as a HAND therapeutic.

#### Cannabinoids

The endocannabinoid system is a complex signalling system comprised of lipid molecules, enzymes and G-protein coupled receptors responsible for mediating a wide range of effects in organisms such as modifying neurotransmitter release, modulating learning and memory, regulating food intake and modulating inflammation and pain reception [313]. These effects are mediated through the activation of cannabinoid receptor 1 (CB1) and cannabinoid receptor 2 (CB2), the latter of which is primarily expressed in the cells of the immune system, including microglia [314, 315]. Synthetic cannabinoid agonists have been evidenced to yield beneficial effects in animal models of numerous neurodegenerative diseases such as Parkinson’s disease, multiple sclerosis and Huntington’s disease [316–318], thus cannabinoids may have similar effects in models of HAND.

Numerous in vitro studies have investigated the potential of cannabinoids in HIV-1 induced neuropathology. For example, using an in vitro co-culture composed of human brain microvascular endothelial cells and human astrocytes, synthetic cannabinoids ACEA and CP55,940 were investigated for their protective effects against gp120 [319]. ACEA and CP55,940 preserved the permeability of the endothelial cells, protected dysregulation of the tight junctions, maintained Ca^2+^ intracellular concentrations, and reduced monocytes transmigration, all of which were affected by gp120 exposure [319]. In another in vitro study, the synthetic CB1/CB2 agonist WIN55,212-2 protected human neurons that were exposed to gp120. Using dopamine transporter suppression as a measure of cellular injury, treatment with WIN55,212-2 blocked the downregulation of dopamine transporter activity and prevented neuronal apoptosis [320]. These effects appear to be primarily mediated by CB2 as the protective effects of WIN55,212-2 were abolished by CB2 antagonist co-treatment, whereas, co-treatment with CB1 antagonist was not sufficient in abolishing the protective effects [320]. WIN55,212-2 and the endocannabinoid anandamide displayed evidence of preventing Tat-induced decreases in GABAergic postsynaptic currents (frequency and amplitude) in murine prefrontal cortices [321]. Interestingly enough, this outcome was implied to be primarily through interaction of CB1 and not CB2, as additional treatment with a CB1 antagonist prevented the occlusion of Tat-mediated GABAergic signalling but the addition of a CB2 antagonist did not [321]. Furthermore, an in vitro study using primary human and murine neural progenitor cells exposed to gp120 showed that treatment with CB2 agonist AM1241 protected against toxicity and apoptosis [322].

In vivo studies investigating cannabinoids in the context of HAND treatment exist but are limited. Gorantla and colleagues tested the immunoregulatory effects of the synthetic CB2 agonist Gp1a in the HIVE mouse model [323]. Treatment with the CB2 agonist resulted in decreases in TNFα expression and microglia activation in the brain as well as a reduction of CCR5 expression in CD4+ cells in the spleen [323]. Another study which used gp120 transgenic mice showed that administration with the CB2 agonist AM1241 increased the number of neurons, neuroblasts and cells positive in markers of proliferation (BrdU, PCNA) in the hippocampi of these mice [322]. Cannabinoid treated gp120 transgenic mice also had decreased instances of astrogliosis when compared to vehicle treated mice [322].

Cannabinoids may have a place in treating HAND. As their exact mechanism in immunomodulation is still not yet fully understood, the details of their role cannot be fully described. There seems to be some inconsistency over which isoform of the cannabinoid receptors (CB1 or CB2) is critical to the immunomodulation of cannabinoids in HAND. Evidence does seem to favour CB2 as being the more important receptor as Purohit and colleagues have written a review focusing on CB2 and how its activation affects HAND [324]. Cannabinoids have also been reported to have antiviral activity and a study demonstrated their ability to inhibit HIV-1 replication in macrophages via activation of CB2 [325]. Studies have demonstrated the beneficial effect that cannabinoids have on GABAergic and dopaminergic signalling, two pathways whose dysregulation is associated with HAND [326–330], which further implicates their therapeutic potential. Continued study of this class of compounds is warranted as the current available data is encouraging.

#### Paroxetine and fluconazole combination

Fluconazole is an approved oral anti-fungal medication whereas paroxetine is a selective serotonin reuptake inhibitor. In 2014, Meulendyke and colleagues screened 2000 FDA approved drugs and natural compounds for neuroprotection against gp120 and Tat neurotoxicity and found the molecules fluconazole and paroxetine to be the most promising candidates [331]. This group then demonstrated that once daily oral treatment with combined fluconazole and paroxetine (FluPar) in SIV-infected rhesus macaques reduced CSF NFL concentrations, accumulation of amyloid precursor proteins and prevented CaMKIIα loss in the frontal cortex [331]. Despite the favourable neuroprotective effects, FluPar did not inhibit viral replication in the brain, CSF or periphery and had little effect on inflammatory markers such as CCL2 and IL-6 in the CNS [331]. Although this study did not show FluPar’s control of SIV infection, it did establish proof of concept of FluPar’s beneficial neuroprotective properties.

As both compounds are already approved for use in humans, a double-blind placebo controlled clinical study testing the efficacy FluPar in attenuating HAND was published shortly after the initial pre-clinical work. The parameters for this clinical trial were as follows: HIV+ patients with cognitive impairment were randomly assigned to placebo, fluconazole, paroxetine, or combination fluconazole and paroxetine treatment for 24 weeks and underwent neurological tests (NPZ8 and the Global Deficit Scale [332]. Participants receiving paroxetine had improved scores in tests measuring reaction time, verbal fluency, visual attention and task switching, with an overall improvement in NPZ8 summary scores but had lower performances in letter number sequencing tests when compared to participants not receiving paroxetine [332]. Paroxetine also had little effect on cellular stress, inflammatory and neuronal damage biomarkers. In contrast, fluconazole treatment, did not show any improvements on cellular stress markers or cognition [332].

Very much like meloxicam, fluconazole showed promise in treating HAND in an SIV macaque model but failed to prove efficacy in human trials. However, paroxetine still may have a significant role in achieving a viable HAND therapeutic as it is well tolerated in humans and showed encouraging data on improving cognition in HIV+ patients [332].

#### Chloroquine

Chloroquine is an FDA approved antiparasitic drug for the treatment of malaria. It disrupts the ability of the malarial parasite to metabolize toxic heme proteins, thus leading to an internal build up of the molecule and ultimately resulting in death [333]. Chloroquine also has mild immunosuppressive effects by inhibiting thiamine uptake via the SLC19A3 transporter [334]. In its treatment for rheumatoid arthritis, its activity reduces lymphocyte proliferation, antigen presentation in dendritic cells, ROS production by macrophages, enzyme release by lysozymes and inhibits phospholipase A2. It is these demonstrated effects that calls into question chloroquine’s potential in treating HIV-associated brain inflammation.

In a 2014 animal model of HIV-induced brain inflammation, rats were intracerebroventricularly injected with gp120 while being pretreated with interparietal minocycline, simvastatin or chloroquine. Upregulation of IL-1β and iNOS was attenuated in the frontal cortex, striatum and hippocampus of the animals pretreated with chloroquine while secretions of IL-1β in the CSF were reduce [64].

Although animal studies with chloroquine and HIV induced brain inflammation appear to be promising, clinical trial assessments have demonstrated no benefit in HIV replication, as well as insignificant immune-dampening properties. HIV+ patients undergoing chloroquine treatment for 24 weeks experienced no change in T-cell activation, absolute counts of CD8+ and CD4+ cells and no reductions in plasma concentrations of cytokines including IFN-γ, TNF-α, IL-1β, IL-6, IL-7, IL-8, IL-12 and IL-13 [335]. A separate randomized trial not only established chloroquine’s inability to reduce CD8+ cell activation in HIV+ patients not on ART, but also found that it exacerbated CD4+ cell count decline and increased HIV replication [336].

## Conclusion

The advent of cART has helped millions of people with HIV-1 and allowed for the management of their infection. Despite cART efficacy, neurological disorders still persist in up to half of those living with HIV-1. Diagnosis of HAND requires exhaustive efforts, and the lack of viable biomarkers increases the challenge of properly identifying affected individuals. Several factors can be associated with the persistence of HAND such as continuous brain inflammation caused by low level HIV-1 replication in cellular reservoirs such as microglia, secretion/shedding of viral proteins and poor penetration of ARVs across the BBB. In order to overcome this pressing issue in such a vulnerable subset of the general population, researchers have employed the use of various animal models in order to further understand the pathology and test pharmacological agents in the pursuit of achieving an effective treatment for HAND. Animal models such as SIV infected macaques and mice treated with chimeric viruses have been useful for evaluating drug candidates in a preclinical setting. The ongoing research has led to promising discoveries such as the efficacy of exogenous IFNβ, maraviroc, and PPARγ agonists. Further studies are still required for such pharmacological entities in order to reach definitive conclusions on their possible use in the clinic.

## Data Availability

Confirmed.

## Abbreviations

AIDS: Acquired immunodeficiency syndrome
ADC: AIDS dementia complex
ALCAM: Activated leukocyte cell adhesion molecule
AMPA: α-Amino-3-hydroxy-5-methyl-4-isoxazolepropionic acid
ARV: Antiretroviral
ANI: Asymptomatic neurocognitive impairment
BBB: Blood–brain barrier
BCSFB: Blood–cerebrospinal barrier
BLT: Bone liver thymus
CCR(*n*): C-C chemokine receptor type(*n*)
CCL(*n*): C-C motif chemokine ligand(*n*)
CNS: Central nervous system
CSF: Cerebrospinal fluid
CD(*n*): Cluster of differentiation(*n*)
cART: Combined antiretroviral therapy
C3: Complement component 3
CXCL10: C-X-C motif chemokine ligand 10
CPE: CNS penetration effectiveness
CXCR4: C-X-C motif chemokine receptor 4
COX-2: Cyclooxygenase-2
DNA: Deoxyribose nucleic acid
dCA: Didehydro-cortistatin A
DMF: Dimethyl fumarate
EAAT(*n*): Excitatory amino acid transporter(*n*)
FIV: Feline immunodeficiency virus
GFAP: Glial fibrillary acidic protein
gp120: Glycoprotein 120
HAD: HIV-associated dementia
HAND: HIV associated neurocognitive disorders
HIVE: HIV encephalitis
HIV: Human immunodeficiency virus
iNOS: Inducible nitric oxide synthase
INFAR(*n*): Interferon alpha–beta receptor
IFNα: Interferon α
IFNβ: Interferon β
IL-(*n*): Interleukin-(*n*)
IL-1β: Interleukin-1β
IL2R: Interleukin-2 receptor
UNAIDS: Joint United Nations program on HIV/AIDS
JAM-A: Junctional adhesion molecule A
MAP-2: Microtubule-associated protein
MND: Minor neurocognitive disorder
MDM: Monocyte derived macrophages
NFL: Neurofilament light chain
NMDA: *N*-methyl-d-aspartate
NF-κB: Nuclear factor kappa-light-chain-enhancer of activated B cells
PPARgamma: Peroxisome proliferator-activated receptor gamma
qPCR: Quantitative polymerase chain reaction
SCID: Severe combined immunodeficiency
SIV: Simian immunodeficiency virus
Tat: Trans-activator for transcription
TNFα: Tumor necrosis factor α
Vpr: Viral protein R
